# *Pseudomonas* 2019 meeting report

**DOI:** 10.1099/jmm.0.001208

**Published:** 2020-06-03

**Authors:** Kim R. Hardie, Kalai Mathee, Herbert P. Schweizer, Lars EP Dietrich, Martin Welch, Teresa de Kievit, Dao Nguyen, Maia Kivisaar, Ajai A. Dandekar, Diane McDougald, Craig Winstanley

**Affiliations:** ^1^​ School of Life Sciences, Biodiscovery Institute, University Park, University of Nottingham, Nottingham, NG7 2RD, UK; ^2^​ Herbert Wertheim College of Medicine, Florida International University, USA; ^3^​ Department of Molecular Genetics & Microbiology, College of Medicine, Emerging Pathogens Institute, University of Florida, Gainesville, FL 32610, USA; ^4^​ Department of Biological Sciences, Columbia University, New York, NY 10027, USA; ^5^​ Department of Biochemistry, Tennis Court Road, University of Cambridge, Cambridge, UK; ^6^​ Department of Microbiology, University of Manitoba, Winnipeg, Canada; ^7^​ Department of Medicine, McGill University, Montreal, Canada; ^8^​ Institute of Molecular and Cell Biology, University of Tartu, Tartu, Estonia; ^9^​ Department of Microbiology, University of Washington, Seattle, WA, USA; ^10^​ The Singapore Centre for Environmental Life Sciences Engineering, Nanyang Technological University, Singapore; ^11^​ The ithree Institute, The University of Technology Sydney, Sydney, Australia; ^12^​ University of Liverpool, Liverpool L69 7BE, UK

**Keywords:** 2019, conference, meeting, *Pseudomonas*, report

The *Pseudomonas* meeting is held every 2 years at different locations across the world, and presentations cover a broad range of species within the genus that includes both plant and animal pathogens as well as biotechnological exploitation of this family of Gram-negative bacteria. This year, between 22 and 26 July, the seventeenth *Pseudomonas* meeting (https://pseudomonasconference.com/) was held at Pullman Kuala Lumpur Bangsar Hotel, Malaysia. Organization was Chaired by Dr Kalai Mathee (Editor-in-Chief, *Journal of Medical Microbiology*/Professor, Florida International University), and was a truly collaborative effort involving an International Organizing Committee, a Scientific Organizing Committee and a local Organizing Committee.

The Conference was attended by 185 people representing 31 countries, with the largest contingent coming from the USA. The Microbiology Society supported the travel of five speakers/organizers and poster awards. The major sponsor, the American Society for Microbiology provided funds to cover the registration of three speakers, four poster presenters, among other expenses. To kick-off proceedings, the delegates were able to provide support to the local community by donating books for a school library. It was an honour to see a selection of students (aged from 9 to 12: M. Tamilalakgy, M. Mithrashinie, P. Sanmukavannan and Mohamed Zedaan) and teachers (Sarojini, principal, Munusamy and alumni chair, Vijay) attending the first scientific session, and the delight they had in the books they received. A legacy such as this is rarely achieved by scientific conferences, and nicely linked to a pre-conference address highlighting the urgent need for microbiology literacy in society delivered by Pablo Ivan Nikel. Ken Timmis was the leader of this initiative and founder of the *Pseudomonas* conference 35 years ago in 1986. Pablo called all the audience to become an active proponent of this initiative which has been outlined in the published editorial https://onlinelibrary.wiley.com/doi/full/10.1111/1462-2920.14611.

The plenary speaker, Professor Shahriar Mobashery (University of Notre Dame, USA), gave an insightful account of his research in cell-wall recycling. The keynote speakers (Professors Dawn Arnold, University of the West of England, Bristol, UK; Joanna Goldberg, Emory University, USA; Iain Lamont, University of Otago, New Zealand; and Mark Wilcox, University of New South Wales, Australia) respectively covered a persistent reservoir of genomic islands in *Pseudomonas syringae *; temperature regulation in *Pseudomonas *; siderophores, signalling and sociobiology; and the NLRP3 inflammasome in keratitis. There were more featured speakers than it is possible to mention individually, but their talks covered aspects of signalling, resistance and novel antibacterials, host-pathogen interaction, metabolism and physiology, harnessing the potential of *Pseudomonas* species, evolution and coevolution, gene regulation and genomics, and multi-species interaction.

Some highlights of the science that covered topics destined for high-profile publication, or that cover technical applications likely to have a major impact on the field of *Pseudomonas* include the following.

## Imaging approaches

- A method harnessing the ability to tether a flagellum to watch the direction of rotation and investigate the regulation of the motor switch by c-diGMP, was presented eloquently by Zhao-Xun Liang (Singapore).
- Abby Kroken (USA) provided insights into the pathogenesis of *P. aeruginosa* corneal infections using the very latest imaging approaches with a mouse mini-contact lens model. These analyses, accompanied by some truly jaw-dropping graphics, illustrated with unprecedented spatial resolution how the pathogen interacts with the epithelial layer in infected corneas, and in particular, how the bacteria manage to cross the epithelial barrier and invade the underlying host tissue.

## Multispecies interactions

- The interactions between the nematode *Caenorhabditis elegans* and *P. aeruginosa* was presented by Varsha Singh (India) from the interesting angle of the nematode, which can sense volatile secondary metabolites enabling it to sniff and avoid *Pseudomonas *. Natalia Kirienko (USA) also presented her work using the *C. elegans-P. aeruginosa* infection model to study the interplay of innate host defences and bacterial virulence. She found that mitophagy, mitochondrial surveillance and proteostasis are host defences involved in mitigating the virulence activity of the siderophore pyoverdine. Small molecules that interfere with pyoverdine function can mitigate the virulence of *P. aeruginosa* infections. Little is known about polymicrobial interactions in complex natural communities, and Rolf Kummerli (Switzerland) examined a collection of fresh water and soil communities. Focusing on phenotypes involved social interactions, such as pyoverdine and protease production, they observed a spectrum of cooperative and competitive behaviours, which were important determinants of the community composition and stability. Further demonstrating the utility of the nematode infection model, Teresa De Kievit (Canada) discussed the regulation of PNAG, an exopolysaccharide that allows biofilm formation on the nematode *C. elegans* by *P. brassicacearum* DF41, a biocontrol agent. Her results suggest a role for the Gac regulatory system and small non-coding RNAs that bind RsmA protein.
- Polymicrobial interactions were extended to include viral-* Pseudomonas* co-infections by Jennifer Bomberger (USA), which may be a future consideration for many studies looking at growth, metabolism and biofilm formation, before being moved into the realms of Artificial Intelligence by Cedric Load (Netherlands). Another speaker who mentioned machine learning was David Baltrus (USA), who suggested it as a way to map genes and their activity through evolution, as part of his interesting discovery of off-target killing by ‘tailocins’, bacteriophage-derived bacteriocins. The tailocins of *P. syringae* provide a mechanism for bacteria to compete with closely related but distinct strains of the same species. He showed data that two specific regions of the *P. syringae* tailocins are prone to extensive recombination and mutation. These recombination-prone areas of the tailocin provide a mechanism that confers novel specificities on the tailocins, including the potential to kill other species altogether.

## Antimicrobial development and resistance

- Lars Dietrich (USA) presented his work recently published in *Nature Communications*, which includes his elegant study of antibiotic tolerance and metabolic heterogeneity within *P. aeruginosa* biofilms. His fascinating talk showcased the state-of-the-art methods that have established that colony biofilms contain an oxygen gradient. With depth, an oxygen-limited zone arises, in which phenazines become progressively more reduced by specific respiratory complexes. Phenazine reduction supports metabolic activity deep in the biofilm, which contributes to bacterial survival and antibiotic tolerance. These types of studies provide insights into how bacteria survive in inhospitable environments and clues as to how factors governing the underlying catabolic pathways could perhaps be exploited as novel drug targets.
- Christopher Evans (USA) followed this up with a map of sigma factor activity within biofilms, and Lisa Juliane Kahl (USA) with the effect of light and dark on metabolic patterns. Dao Nguyen (Canada) spoke about the mechanisms underlying antibiotic tolerance in stationary phase. Her group discovered that accumulation of cyclopropane fatty acids (CFA) contributes to stationary phase multidrug tolerance. Impairment of this tolerance by deletion of CFA synthase results in increased membrane permeability and thus increased penetration of diverse antibiotics such as β-lactams and fluoroquinolones. The premise, therefore, is that CFA synthase inhibitors could be identified that may potentiate antibiotics currently used in the treatment of *P. aeruginosa* infections. Another aspect of altered physiology relevant to biofilm formation was presented by Claire Kirkpatrick (Denmark) who elaborated on the roles that SOS response regulators and the HigAB toxin-antitoxin system play in *P. aeruginosa* persister cell formation. Using a combination of mutant, gene fusion and SOS regulator overexpression studies, the work by her laboratory indicated that a complex interplay between at least one of three LexA-type SOS response regulators and the HigAB toxin-antitoxin system may have a global influence on antibiotic-induced persister cell formation. Of potential relevance to this was the talk by Scott Rice (Singapore) who described the Pf4 prophage in *P. aeruginosa,* which mediates stress resistance, virulence and biofilm formation. The Pf4 phage is linked to cell death with biofilm microcolonies and small colony variants. Transcriptomic analysis showed that the phage regulated virulence and biofilm genes. One gene in the phage genome had homology to phage immunity proteins and was shown to bind a putative phage replication regulatory region and to prevent phage infection. ChIPSeq data also showed that the immunity protein bound and regulated a number of host genes. Crystal structure analysis of wild-type and a non-functional variant of the immunity protein suggest how superinfection is regulated.
- Martin Welch (UK) presented an elegant fusion of chemistry and crystallography to show how an understanding of the biology of short-chain fatty-acid catabolism can be exploited to discover new antimicrobials.
- Tim Wells (Australia) presented a promising treatment approach for lung infections using inhibitory antibodies. His remarkable clinical observation was that in patients with extremely high titres of O-antigen, specific IgG2 antibodies display impaired serum-mediated killing of *P. aeruginosa *. Not surprisingly, this is accompanied by increased infection severity. Most remarkably, a selection of patients undergoing plasmapheretic removal of these inhibitory IgG2 variants immediately benefited from the treatment, suggesting that the approach may have therapeutic benefit for some patients in the longer-term.

## Host-pathogen interactions

- Bryan Swingle (USA) described how *P. syringae* downregulates flagellin expression, helping to minimize plant immune system activation.
- Rob Jackson (UK) described the use of dual RNAseq and other approaches to study the regulation of toxins produced by *P. poae *, of the *P. fluorescens* complex, with activity against aphids. He focused on *Pseudomonas* toxins that are able to kill aphids as a means of biological control. Application of the bacterium to plants before aphid colonization led to a 65 % reduction in aphid infestation. Through RNA sequencing, very few changes in aphid gene expression were observed; however, a large number of bacterial genes (1342) showed altered expression in the bacterial-aphid colonization assays. Five different insecticidal toxins are produced by this bacterium, all of which contribute to aphid killing to some degree.

## Signalling systems

- Benoit-Joseph Laventie (Switzerland) described an interesting reporter for c-di-GMP that indicates differentiation between mother and daughter cells after division, and suggests that bacteria may touch surfaces, divide and some will leave. Investigation of similar signalling by Paul Williams (UK) has supported development of novel biosurfaces that protect catheters from infection in patients. Paul’s refreshingly different approach to inhibiting biofilm formation on biomaterials is based on the rather simple but clever premise that in the age of multidrug resistance, efforts aimed at discovery of materials that are inherently resistant to biofilm formation would be more fruitful than traditional approaches that are focused on incorporation of antimicrobial agents into biomaterials. Screens for biofilm-resistant materials and pre-clinical studies led to discovery and characterization of a polymer on which *P. aeruginosa* biofilm development stalls. An optimized polymer is now in clinical trials for prevention of catheter-associated urinary tract infections.

## Omics methodologies

- Methods were openly promoted by Stephen Lory (USA), who described GRIL-Seq as a way to identify regulatory small RNAs, and offered to share it widely as he has generously done with previous resources and protocols. The method is based on preferential ligation of sRNAs to the ends of base-paired targets in bacteria co-expressing T4 RNA ligase, followed by sequencing to identify the chimaeras. For example, several new targets of PrrF1, an iron-regulated sRNA, were identified by using this method. In addition to regulating mRNA stability and translation initiation, several novel mechanisms of gene expression regulation were discovered (e.g., the action of mRNA-derived short transcripts in counteracting the regulatory effects of other RNAs).
- Aindrila Mukhopadhyay (USA) discussed the use of functional genomics tools to analyse biosynthetic pathways in order to exploit *Pseudomonas* for bioconversion.

Additional top-class research was presented in the ‘Metabolism and Physiology’ session, highlighting the impressive metabolic versatility of pseudomonads, which allows for their swift adaptation to diverse environments, and creative ways to exploit this versatility in drug design and metabolic engineering. Pablo Ivan Nikel (Denmark) provided an impressive overview of advances made in using *P. putida* as a chassis for creating cellular factories, by streamlining or even exchanging elements of central metabolism with those from other organisms. Christoph Wittmann (Germany) presented an elegant, ‘tour de force’ approach in which knowledge about metabolic pathways is used to create a multistep procedure for converting lignin, the second most abundant biopolymer on earth, into muconic acid (MA). Perhaps some of the synthetic biology applications showcased by Esteban Martinez-Garcia (Spain) in *P. putida* might be of use for this. A set of genetic tools that allowed manipulation of the bacterial genome was described. Findings demonstrated that combining ssDNA recombineering using mutagenic oligonucleotides and a *Pseudomonas* phage recombinase enabled DNA changes ranging from single nucleotide substitutions to large deletions. Moreover, coupling this approach with CRISPR/Cas9 allows for strong counter-selection of wild-type sequences, enabling easy selection of clones with the desired changes. Creation of a variant wherein non-essential outer membrane protein genes were deleted demonstrated the utility of this exciting technology.

An offered talk, given by Mayuree Fuangthong (Thailand) focused on tRNA, a molecule perhaps many of us take for granted. She described a novel role of tRNA modification enzyme TrmB in oxidative stress response in *P. aeruginosa *, a stress response which is important in survival of *P. aeruginosa* during infection. TrmB is a methyltransferase that catalyses guanine 46 methylation in the tRNA variable loop (M^7^G46 modification). The involvement of *trmB* in oxidative stress tolerance was discovered by screening a mutant library of tRNA modification genes. Subsequent studies using codon reporter assays revealed that *trmB* is specifically required for the efficient translation of Phe- and Asp-enriched mRNAs, including catalase genes *katA* and *katB*. Compared to the full *P. aeruginosa* genome, these two catalase genes were enriched in Phe/Asp codons. The positive correlation between the level of M^7^G46 modification and the elevated catalase expression in the presence of hydrogen peroxide was demonstrated, thereby suggesting the enhancement of translation efficiency of catalase genes by TrmB via a biassed codon usage mechanism.

As a final point, it was refreshing to hear about the myriad selective pressures and drivers of evolutionary change among both environmental and clinically relevant pseudomonads. Craig Winstanley (UK) described evolution of *P. aeruginosa* multidrug resistance plasmids, Maia Kivisaar (Estonia) described pollutant-driven evolution of new phenotypes in *P. putida *, Russell Poulter (New Zealand) described horizontal gene transfer in the kiwifruit pathogen *P. syringae* pv actinidiae, and Lea Girard (Belgium) discussed nonribosomal peptide synthetase (NRPS) evolution in *Pseudomonas* genomes that are involved in the production of biosurfactant cyclic lipopeptides. Together, these talks emphasized the diversity among the species of the genus and the diverse environments in which the bacteria can thrive and adapt.

The excellent standard of the offered oral presentations made it a very tough decision to select the winners of the prizes, as did the exciting science and standard of presentation represented in the posters. To provide the opportunity for Early Career Researchers (ECRs) to be fully involved in the conference, every session had an ECR co-chair, and all of the poster presenters were given the opportunity to present a 1.5 min oral Flash presentation to entice the other delegates to find out more about their work during the poster sessions. Amazingly, the chairs of the Flash Poster presentation (Kim Hardie and Christoph Wittmann) were able to impose strict timing, and with the help of a few speedy presenters, fit 100 presentations into 2 h, allowing enough time for the audience to stretch their legs intermittently with some fun exercises and jokes.

All awards came with cash, certificates and a *Pseudomonas* artwork by Professor Kalai Mathee, and the winners are listed below:

The postdoctoral oral presentation/early career scientist awards: sponsored by **The Federation of European Microbiological Societies** and **Academic Nexus for Global Scholastic Activities (ANGSA**)

- Third prize: **Dr Stephan Dolan** of University of Cambridge, UK.
- Second prize: **Dr Maxim Kostylev** of University of Washington, USA.
- First prize: **Dr Benoit-Joseph Laventie** of University of Basel, Switzerland.

The postdoctoral poster awards: sponsored by the **Journal of Medical Microbiology**.

- Third prize: **Dr Aneta Agnieszka Bartosik** of Polish Academy of Sciences, Poland.
- Second prize: **Dr Irene Bianconi** of University of Trento, Italy.
- First prize: **Sophie Howard** of Imperial College, UK.

Oral Presentation Awards to graduate students: sponsored by the **Journal of Medical Microbiology**

- Third prize: **Gabriela Purtschert** of University of Zurich, Switzerland.
- Second prize: **Lisa Juliane Kahl** of Columbia University, USA.
- First prize: **Corrie Belanger** of University of British Columbia, Canada.

Poster awards to graduate students: sponsored by **ANGSA**.

- Third prize: **Carolina Paiva** of University of Nottingham, UK.
- Second prize: **Hyeon-Ji Hwang** of Pusan National University, South Korea.
- First prize: **Stephane Pont** of University of Grenoble Alpes, France.

The conference attendees enjoyed a cultural visit that included a Buddhist temple, a demonstration of the traditional art of Batik, a Hindu temple and a Mosque, as well as a traditional meal served on banana leaves and heralded by a procession of traditional music. There was also an interesting injection of novel art at the conference, with the bespoke conference icon, one-off printed scarfs that incorporated *Pseudomonas *, and even a couple of specially designed cocktails. Prize winners were lucky enough to be presented with original art designed by the Conference Chair, Dr Kalai Mathee.

The overriding memory of this meeting for all delegates is likely to be the fantastic and plentiful food that was provided throughout the conference. With all tastes and cultures being represented (Malay, Indian, Chinese, Western), it is hard to imagine that anyone went hungry. This will be married with the networks of collaborators and friends that will have been initiated and reinforced by the discussions over the meals, scientific sessions and cultural experiences. Let us hope that some ECRs secured future employment in the field!

The next meeting is scheduled to take place in Atlanta, USA in 2021, so look out for further details about the interesting science presented by high-profile speakers.

